# Evaluating emotional labor from a career management perspective

**DOI:** 10.3389/fpsyg.2022.1093723

**Published:** 2023-01-16

**Authors:** Yunhong Hu, Wei Tu, Li Zhou, Xin Wu, Qi Yan

**Affiliations:** ^1^Department of Urban and Rural Planning, School of Landscape Architecture, Nanjing Forestry University, Nanjing, China; ^2^Department of Tourism, School of Tourism and Social Administration, Nanjing Xiaozhuang University, Nanjing, China

**Keywords:** emotional labor, organizational support, career competences, career commitment, China

## Abstract

Emotional labor claims its significance as the key indicator both of the psychological health of contemporary employees, and the productivity of service-based businesses depending upon genuine emotional input of employees. By far, research on emotional labor of employees in an organizational context is still lacking. This study aims to explore the relationships among emotional labor, organizational support, career competences and career commitment to investigate how emotional labor interacts with the organizational context and affects the career management of the employee. Data were collected from a sample of 387 frontline employees working at two luxury hotel brands in China. Structural equation modeling (SEM) was utilized to estimate the relationships among the constructs. It is demonstrated by the findings that organizational support mediates positively on emotional labor, which exerts positive influences on career competences and career commitment. Sound handling of emotional labor, boosted by a supportive organizational environment, has been ascertained to positively predict long-term career paths of the employees at the company. This study provides insights into how the tourism and hospitality industry can optimize the functions of emotional labor for in enhancing service quality and customer satisfaction, as well as promoting the psychological well-being of the employees.

## Introduction

The salience of emotional labor has been long established in the tourism and hospitality industry (Pugh, 2001; Wong and Wang, 2009). For instance, Disneyland always brands its employees as performers and actors/actresses, while in the hotel sector the title of ‘smile ambassadors’ is the norm rather than exception. Given the inherent service features of the tourism and hospitality industry such as the intangibility of the products, the emphasis on the experiential values that the customer can achieve, and the high incidence and complexity of customer-employee interactions, emotional labor is a key requirement for tourism and hospitality jobs at all levels (Lucas and Deery, 2004). It has been suggested by Yan et al. (2012) that in the tourism and hospitality arena, emotional labor should assume no less significance than the work input by the employees in physical and intellectual aspects. Taking cognizance of the growing penetration of ‘theaterization’ into the tourism and hospitality industry, emotional labor is expected by Kim (2008) to take the central stage in real terms.

From a managerial perspective, therefore, emotional labor should be addressed and cultivated to such an extent that it cannot only enhance the job performance of the employees, but also contribute to their psychological well-being. Besides, emotional labor should be regarded as an essential aspect for the realization of the corporate objectives of the business (Morris and Feldman, 1997). For many service-based enterprises, emotional labor can become the factor distinguishing their core competitiveness (Kim, 1998). Moreover, with the growing popularity of the internal customer paradigm, positive emotional labor is posited by Zapf and Holz (2006) to be the prerequisite for sound organizational behaviors, which directly leads to the successful operation and management of the tourism & hospitality enterprises. Meanwhile, emotional labor of employees has been established as closely related to their job satisfaction with the workplace and long-term commitment to it, with employees who are burnt out in their emotional labor more likely to display emotional dissonance and detachment, leading to job exits and career aversions (Tosten and Mustafa Toprak, 2017; Asumah et al., 2019). In this sense, emotional labor guarantees investigation from the perspective of career management for the sustainable operation of the business (McGinley et al., 2019).

While it has been widely recognized that emotional labor can be closely supervised and controlled by employers (Pizam, 2004; Bolton, 2005; Chu and Murrmann, 2006), by far most research efforts on it have been mostly concentrated on the employee side such as their job autonomy, job satisfaction, emotional dissonance & exhaustion, psychological well-being etc. (Kim and Han, 2009; Chu et al., 2011; Gursoy et al., 2011). This study intends to tackle the current research paucity of addressing emotional issues of employees from the angle of career path, through investigating the relationships between emotional labor and the significant managerial factors of perceived organizational support, career competence and career commitment in tourism and hospitality context. The specific research objectives of this study are three-fold: first, to explore and propose the conceptualization of emotional labor, perceived organizational support, career competence and career commitment for hospitality employees; second, to assess the interrelationships among the proposed concepts; third, to generate theoretical as well as practical insights that can serve as references for agile leadership in caring for emotional ramifications of employees in contemporary organizations.

## Literature review

### Emotional labor

Grandey (2000) semantically conceptualized emotional labor as the process of emotion regulation and expression by employees for the realization of organizational objectives. According to this definition, emotional labor is the management of emotions by the employees in the workplace, displaying and adjusting feelings and expressions that are needed to fulfill the requirements of the job (Brotheridge and Lee, 2003). In addition to this value-laden feature, emotional labor is also characterized by the responsiveness from the customers, the normative requirements from the employer as well as its omnipresence among employees at different levels (Lucas and Deery, 2004).

As is suggested by Liu et al. (2004), emotional labor is a concept that is of close relevance but yet not limited to the service sector, with the tourism and hospitality industry no exception here. Actually, one of the earliest examination on emotional labor was under the context of the airline sector, indicating the salience of this issue in the tourism and hospitality industry (Hochschild, 1983). It can be said that the job performance of a tourism and hospitality employee is entirely emotion-oriented (Pizam, 2004). On one hand, the facial expressions and body languages are already integrated into the quality of service provided by the employee to the customer, which can be best illustrated by the recognition of the factors of empathy and assurance as the key dimensions of service quality. On the other hand, emotional labor is dynamic rather than static in the service process, open to instant adjustment and optimization in accordance with the feedback from the customer, so that the service product can be successfully consumed. Since customer satisfaction, attitude and loyalty are all best expressed in emotional forms, the resonance functions of the emotional labor of the employee are more and more cherished and anticipated (Yan et al., 2012). Thus, great stakes are placed on the presentation and management of emotional labor for the enterprise, whether in soliciting new customers or the retention of established ones (Brotheridge and Lee, 2003). Hochschild (1983) provided a classical categorization of emotional labor into three streams of surface acting, deep acting and genuine acting. In surface acting, employees fake their motions through observable features in gestures, expressions and voice tones to align with the requirements of their job; deep acting is concerned with more active involvement of the inner states of the employee in addition to surface acting, through the utilization of mental and psychological maneuvers such as thoughts, memories and images. In other words, the employee would ‘labor out’ the expected emotions in deep acting. The third category of genuine emotion is where the felt emotions are congruent with the expressed ones, as the employee express what he or she experiences in a spontaneous and natural manner. Genuine acting is consistent with the term of ‘naturally felt emotions’ offered by Diefendorff et al. (2005). The existence and effects of different forms of emotional labor indicate the dilemma and even conflicts of ‘feeling rules’ and ‘display rules’ in the work environment of many industries, which may easily lead to emotional dissonance and exhaustion and thereby severe mental and psychological challenges to the employee. For instance, in the service industry, job burnout is usually preceded by emotional exhaustion, which is much more noted at middle and senior managerial levels (Bolton, 2005). Conversely, positive effects of emotional labor can arise from the synergy of felt and display emotions such as job satisfaction, self-efficacy and self-esteem (Chu and Murrmann, 2006).

With regard to factors influencing the generation and outcomes of emotional labor, two sources have been identified, namely, personal and organizational (Lucas and Deery, 2004; Yang and Chang, 2008). From a personal perspective, emotional labor has been discovered to be related to the work experiences, personality as well cultural values of the employee. On this account, it has been postulated by Xu (2007) that some employees may be inherently unfit for the emotion labor required by certain professions. This, nevertheless, does not rule out the emotional efforts that can be wielded by the employee to achieve certain expected emotional outcomes without rendering negative emotional consequences. In addition, with the growing level of diversity in the workforce of the tourism and hospitality industry, the cultural underlying of emotional labor are more and more evident (Yan et al., 2012). From the organizational perspective, the consensus has been reached that emotional labor can be effectively regulated and managed so that the positive effects of emotional labor can be induced. Particularly, effective emotional management can introduce energetic and amusing dynamics into the otherwise monotonous tasks and compensate for the ‘dull aspects’ of the job. Meanwhile, emotional labor can be appropriately addressed through the facilitation of job autonomy, which has been empirically examined by Kim et al. (2007) to make positive contributions to the prevention of emotional dissonance and exhaustion.

### Perceived organizational support

Perceived organizational support can be defined as the perceptions by the employee of the evaluation of work and extent of support from the organization (Allen, 1995). Perceived organizational support reflects the interactions between the organization and employee, with the aim of realization of an interrelationship that is based on reciprocity. Perceived organizational support is derived from social exchange elaborations, and can be embodied in material, psychological as well as emotional terms. In the professional arena, perception by the employee of perceived organizational support should encompass not only expectations for remunerations that match job performance, but also demand for in-time care and necessary help from the employer. Hutchison (1997) stressed that particularly in scenarios of emergencies or tremendous challenge, the values of perceived organizational support in psychological and emotional forms would overweigh those in monetary forms. In this sense, the psychological contracts between the employee and the employer would be consolidated to such an extent that greater trust, loyalty and sense of belonging would be secured for the organization. Generally speaking, perceived organizational support is recorded to be more salient when such support is perceived as spontaneous, interactive and responsive (Settoon et al., 1996).

Eisenberger et al. (1997) identified three major dimensions of perceived organizational support, namely organizational rewards & job condition, supervisory support and fairness. Organizational rewards & job conditions consist of tangible material rewards as well as those intangible benefits like working experiences beneficial for future career development, autonomy in job, sense of security etc. Such organizational endowments are direct indications of organizational commitments to the employee, and help enhance the in-role and out-role behaviors of the employee toward the achievements of corporate objectives (Hutchison, 1997). Supervisory support is usually presented in the forms of attention to the employee, recognition of the employee’s contribution and communications of corporate values. It was demonstrated by Yoon and Lim (1999) that supervisory support is perceived most strongly from the immediate superiors of the employees, which is an apt reflection of the peculiarities of the hotel sector which are characterized by close and dynamic supervisor-employee interactions. The third dimension of procedural justice, which underpins the equity principle in social exchange elaborations, is related to the perception by the employee support as against those of other employees. From rewards & penalties policies to allocation of organizational resources, procedural justice is actively pursued and appreciated by the employee, and is a key determinant of the balance and harmony of interrelationships among the employees.

Vigorous organizational support has been found out to incease job satisfaction of employees (Hutchison, 1997), a favorable outcome that is strongly associated with sound handling of emotional labor (Asumah et al., 2019; McGinley et al., 2019). In view of the above review of emotional labor and perceived organizational support, the first research hypothesis is proposed as follows:

*H1*: There is a positive relationship between organizational support and emotional labor.

### Career competencies

Eby et al. (2003) defined career competencies as the portfolio of work experiences, skills and qualifications that the employee progressively accumulated during the career. As against those attributes relevant to the required job performance of an employee, career competencies address the employee capacities on at a longitudinal, strategic and sustainable level (Arthur et al., 1995). In this regard, career competencies introduce intelligence aspects into the career management of the employee, and aim for the integration of individual and corporate successes (Eby et al., 2003). Especially, career competencies are significant determinants of positive career changes along the career path of the employee. In addition, with growing emphasis placed on systematic and comprehensive career management, career competencies are purported as significant criteria for career progress to managerial and leadership positions (Sturges et al., 2003).

Three branches of career competencies were articulated by Arthur et al. (1995), which are ‘Knowing why’, ‘knowing who’ and ‘knowing how’. Knowing why concerns the motivational aspirations of the employee in self-identification, self-understanding and possibilities exploration. Knowing why also relates to the ability of adaptation to shifting work contexts. Knowing why can be further accounted for by the three variables of career insight, proactive personality, and openness to experience. Knowing who is synonymous with the networks and contacts from within or beyond the organization, the values of which can be exchanged for the employee’s career progress. Knowing who within the organization encompasses normative relationships as well as those with mentoring natures. Knowing who outside the organization, on the other hand, can extend to contacts such as suppliers, consultants and professional acquaintances. The more conventional concepts of career-relevant knowledge and skills fall into the category of knowing how, which is featured by accumulation and foundation (Sturges et al., 2003). Knowing how consists of two inter-related sub-categories of career-related skills and career identity, with the latter serving as the motivator and director of skills accumulation (Eby et al., 2003).

Career competencies in the tourism and hospitality industry have been opined by Tesone and Ricci (2006) to be one of the most comprehensive and demanding in the service sector, given the diversity of customer needs, combination of physical and psychological inputs and potential of cross-cultural conflicts. The requirements of flexibility and personalization that characterize this industry pose further challenges to the cultivation of career competencies in this industry (Christou, 2002). Major competencies that have been identified are positive attitude, communication skills, effective listening as well as willingness to work hard (Chung, 2000; Brownell, 2008), thereby leading to the formulation of the second research hypothesis of this study as follows:

*H2*: There is a positive relationship between emotional labor and career competences.

### Career commitment

Career commitment is purported by Blau (1999) to be an attitudinal measurement of one’s motivation to work in a chosen vocation. In this regard, career commitment can be analogized as a psychological contract between an individual and his/her career, indicating the level of attachment and involvement to the career by the individual, the degree of alignment of individual objectives with organizational ones, as well as the extent to which the career takes the central stage of the individual’s daily life. Meanwhile, another paradigm emphasizes the behavioral aspects of career commitment, focusing on the temporal and monetary costs and benefits that are incurred to the individual arising from the commitment behavior. An employee would conduct a subjective person-organization fit evaluation, and the differences between the exact career peculiarities and the expectations of the employee would determine the outcomes of career commitment (Carson and Bedeian, 1994). Career commitment has merits in reliably predicting such important factors as work pressure, job efficacy and satisfaction. Career commitment was firstly applied in the education sector and has been widely adopted by tertiary sectors to examine and tackle employee absenteeism and quitting (Cohen, 1999).

**Figure 1:**
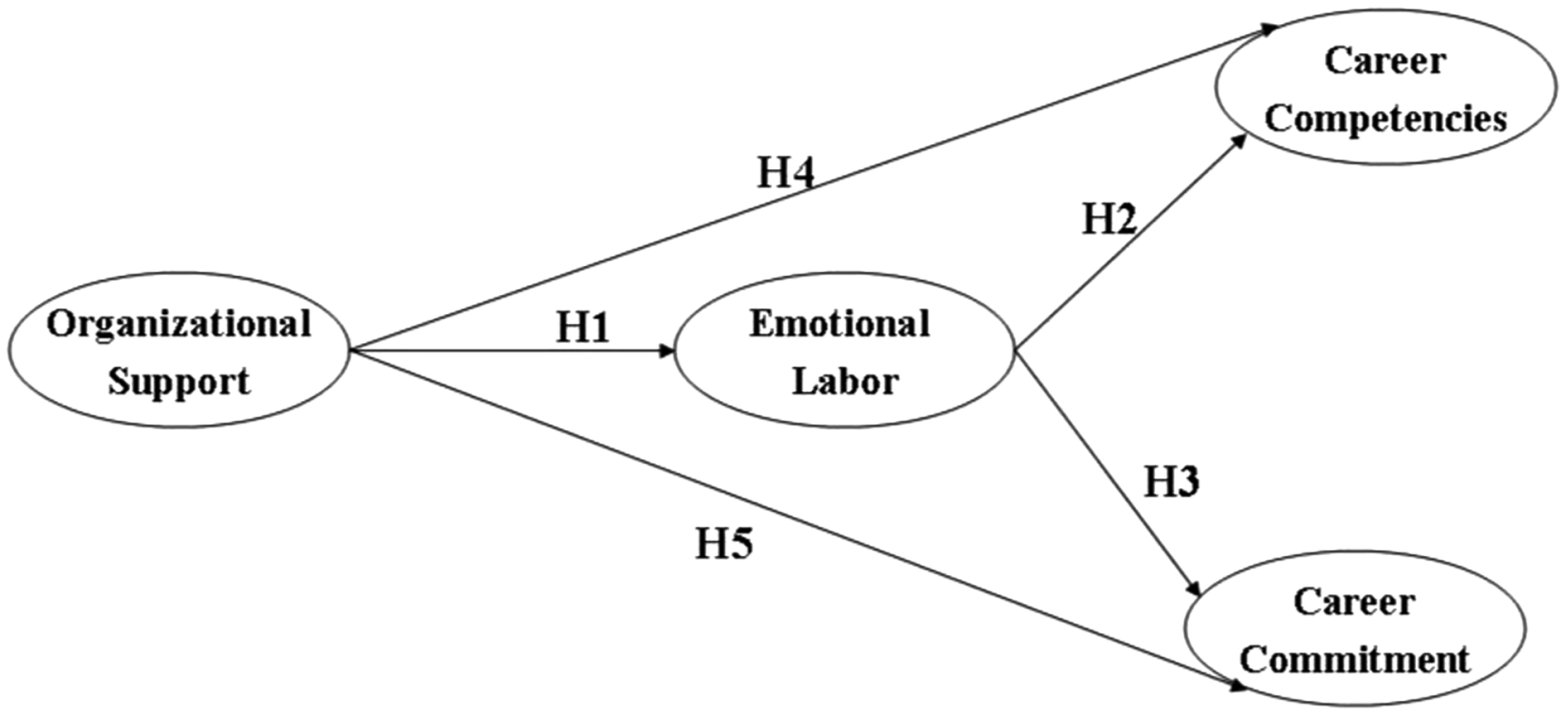
Conceptual framework.

Career commitment is a dynamic concept that is based upon the constant and active interactions between the employee and the career milieu, and may have taken form even before one enters a certain career (Chang, 1999). Cohen (1999) listed out three major factors underlying the emergence of career commitment, namely the predilection for the career, balance of the costs of shifting career and concern over violation of social norms in career change. Respectively, career commitment can be divided into emotional, cost and normative categories. It can be learned from this classification that career commitment can be affected by both intrinsic and extrinsic attributes of the employee, such as demographic features, personality, salary, work environment and work challenges, etc. In the tourism and hospitality contexts, career commitment has been found to be positively related to educational backgrounds, titles, foreign language skills and professional certifications, thereby illustrating the vast spaces that can be explored to enhance the career commitment of tourism and hospitality employees (Leslie and Russell, 2006; Walsh and Taylor, 2007). Therefore, the third to fifth research hypotheses are generated, which are coalesced with the first two hypotheses and presented in the research framework of this study as displayed in Figure 1:

*H3*: There is a positive relationship between emotional labor and career commitment.

*H4*: There is a positive relationship between organizational support and career competences.

*H5*: There is a positive relationship between organizational support and career competences.

## Method

### Scale development

In accordance with relevant literature, emotional labor was measured on a three-dimension scale which included the attributes of surface acting, deep acting and genuine acting. The measurement proposed by Diefendorff et al. (2005) was the primary reference, with attention also paid to the scale developed by Kim and Han (2009) which was of more hospitality features. Altogether, seven, five and two items were proposed for surface acting, deep acting and genuine acting, respectively. Sample items included “In my job, controlling my emotions is very important,” “I more often have an artificial smile than natural smile at work,” “There is a great big difference between my real emotions and expressed emotions” and “I frequently hide my emotions on the job.”

At the same time, the measurement of organizational support was based on the one proposed by Friedman and Greenhaus (2000) with six items, such as “The organization values my contribution to its well-being.,” “The organization really cares about my well-being,” “The organization cares about my general satisfaction at work.,” “The organization takes pride in my accomplishments at work.” Meanwhile, Kong et al. (2012)‘s refined scale of career competences was adapted and adopted, with six items for examination. Sample items were “I seek out opportunities for continuous learning” and “I have a diversified set of job-related skills.” At last, the scale measuring career commitment was refined from that used by Blau (1999), consisting of six items like “If I had all the money I need without working, I would probably still continue in this profession,” “I would recommend a career in the hospitality industry to others” and “I am disappointed that I ever entered this profession.” All the items were measured on a seven-point Likert scale with 1 being “strongly disagree” and 7 being “strongly agree.”

### Data collection and analysis

A self-administered questionnaire was developed and distributed to 450 frontline employees in three five star international-brand hotels in Shanghai, China in mid-2022. The benchmark criteria for the interviewee were set at full time employee with more than 6 months of working experiences. In light of a quite internationalized workforce at the investigated hotels, the questionnaire was designed in both English and Chinese, although the nationality and native language were not solicited given the research purposes of this study. While the survey was being filled, trained interviewers would assist with clarification issues when necessary. Out of the 450 copies of surveys distributed, 406 were returned and 387 were determined to be valid for further analysis after initial examination, recording a response rate of 86%, which reached a satisfactory level for further data analysis.

The data analysis process consisted of two steps. Firstly, a confirmatory factor analysis (CFA) was conducted to investigate the internal validity and reliability of the proposed scale. This stage included the examination of dimensionality and convergent and discriminant validity (Anderson and Gerbing, 1988); secondly, the structural equation model (SEM) was applied to test the fitness of the proposed model and the hypotheses developed early. The computer software of SPSS 15.0 and AMOS 17.0 were employed in data categorization and model evaluation.

### Profile of respondents

The valid interview participants served in front office, housekeeping, food & beverage, recreation and marketing departments of the hotel, with 32.4% indicating intra-or cross-departmental experiences. There were 42.3% males and 57.7% females. The age range of 20–30 years recorded the greatest proportion at 68.0%, followed by 31–40 years (18.2%) and 41–50 years (12.4%). The majority of respondents reported holding a bachelor’s degree (46.7%), followed by secondary school (36.2%), graduate (13.2%) and below secondary school (3.9%). In terms of working experiences, the distribution turned out to be quite even among the categories of 1–5 years, 5–10 years and over 10 years. Particularly, employees who had working experiences at more than two hotels accounted for 52.3% of the total, while 37.5% of the respondents reported to have had worked at other service industries before taking the job at the hotel.

## Results

### Confirmatory factor analysis of individual measurement model

The CFA was conducted to investigate the reliability and then validity of the respective proposed measurements with the other subsamples, with the results manifested in Table 1. Overall, as the composite measures of reliability of the examined variables all stayed above 0.70, satisfying the criteria suggested by Hair et al. (2014), it was found that the items concerned were reliable in indicating their corresponding dimensions and that a satisfactory level of internal consistency within each proposed dimension was achieved. As is stipulated by Byrne (2010), standardized factor loadings with a value of less than 0.5 were deemed insignificant and prone to the possibility of cross-loading. Therefore, 5 items were discarded from the proposed scale of emotional labor due to falling below the level of 0.5, including 2 from the surface acting and deep acting dimensions, respectively, and one from the genuine acting dimension; meanwhile, two items were dropped from the original scale for organizational support, while one item each was voided from the proposed measurements of career competencies and career commitment.

**Table 1:** Confirmatory factor analysis of dimensions of examined factors (*N* = 387).

Construct	Dimensions	Standardized dimension loading	Composite reliability
Emotional labor			0.76
Surface acting			
	Surface acting		0.80
	1. I often pretend to have the emotions I need to show for customers	0.78	
	2. I often fake to customers that I am in a good mood	0.70	
	3. I can create a look of concern for the client when in reality I am not	0.68	
	4. I often put on an act in order to deal with customers	0.66	
	5. Even if I am in a bad mood, I can leave a good impression with the customers	0.66	
Deep acting			
	Deep acting		0.71
	1. I can manage my emotions to help me understand the customers’ perspectives	0.72	
	2. I try to feel the positive emotions I must show to the customers	0.70	
	3. I can separate my feelings enough to deal with tough customers	0.70	
Genuine acting			
	Genuine acting		0. 77
	1. I feel it is difficult not to express my real emotions at work	0.64	
	2. I feel embarrassed for the difference between real emotions and expressed emotions	0.76	
Organizational support			
	Organizational support		0.91
	1. The organization really cares about my well-being	0.71	
	2. The organization takes pride in my accomplishments at work	0.76	
	3. The organization appreciates any extra effort from me	0.92	
	4. The organization shows great concern for me	0.67	
Career competences			
	Career competences		0.78
	1. I have clear career insight.	0.53	
	2. I am open to experience	0.66	
	3. I have career-related skills	0.71	
	4. I seek advice from mentor about my career development	0.70	
Career commitment			
	Career commitment		0.784
	1. I definitely want a career for myself in my current area	0.56	
	2. If I could do it all over again, I would choose to work in this profession	0.62	
	3. If I had all the money I need without working, I would probably still continue in this profession	0.57	

All of the measurement models reported goodness-of-fit indices that could establish an acceptable fit between the model and the sample data: (1) emotional labor measurement model (*x*^2^ = 436.32, *df* = 27, CFI = 0.91, GFI = 0.94, RMSEA = 0.07); (2) organizational support measurement model (*x*^2^ = 373.24, *df* = 22, CFI = 0.93, GFI = 0.91, RMSEA = 0.07); (3) career competences measurement model (*x*^2^ = 321.61, *df* = 25, CFI = 0.93, GFI = 0.92, RMSEA = 0.07); (4) career commitment measurement model (*x*^2^ = 406.97, *df* = 19, CFI = 0.91, GFI = 0.93, RMSEA = 0.07). According to the criteria offered by Hu and Bentler (1998), CFI and GFI values over 0.9 were deemed as acceptable for model fit. Accordingly, the proposed scales were all uniquely related to their respective dimensions and a satisfactory level of convergent validity was obtained. Then, discriminant validity of the scale was assessed to further validate the proposed scale of switching costs. Here, average variance extracted (AVE) analysis (Henseler et al., 2015), was adopted. It was shown by the results as in Table 2 that the square root of the average variance for each of the proposed dimensions was greater than any of the inter-correlations of the dimensions. This analysis therefore gave additional support for the discriminant validity of the proposed scale.

**Table 2:** Correlation and discriminant validity.

Constructs	Mean	Standard deviation	AVE	SA	DA	GA	OS	CMC	CC
Surface acting (SA)	5.047	1.362	**0.690**	**0.789**					
Deep acting (DA)	5.331	1.340	0.628	0.520^**^	**0.792**				
Genuine acting (GA)	5.701	1.267	0.681	0.461^**^	0.567^**^	**0.791**			
Organizational support (OS)	5.092	1.399	0.751	0.488^**^	0.483^**^	0.347^**^	**0.751**		
Career competencies (CMC)	5.155	1.490	0.599	0.421^**^	0.455^**^	0.405^**^	0.515^**^	**0.779**	
Career commitment (CC)	5.081	1.441	0.702	0.379^**^	0.487^**^	0.350^**^	0.452^**^	0.463^**^	**0.838**

^**^Correlation is significant at the 0.01 level (2-tailed). Bold font is square-root of AVE.

The overall measurement model, which recorded the goodness-of-fit indices of *x*^2^ = 1034.77, *df* = 271, CFI = 0.90, GFI = 0.93, RMSEA = 0.07, demonstrated a considerable degree of good fit between the model and the sample data. As the composite measures of reliability ranged from 0.78 to 0.91, a sound fit had been established between the overall measurement model and the data.

### Structural model

**Figure 2:**
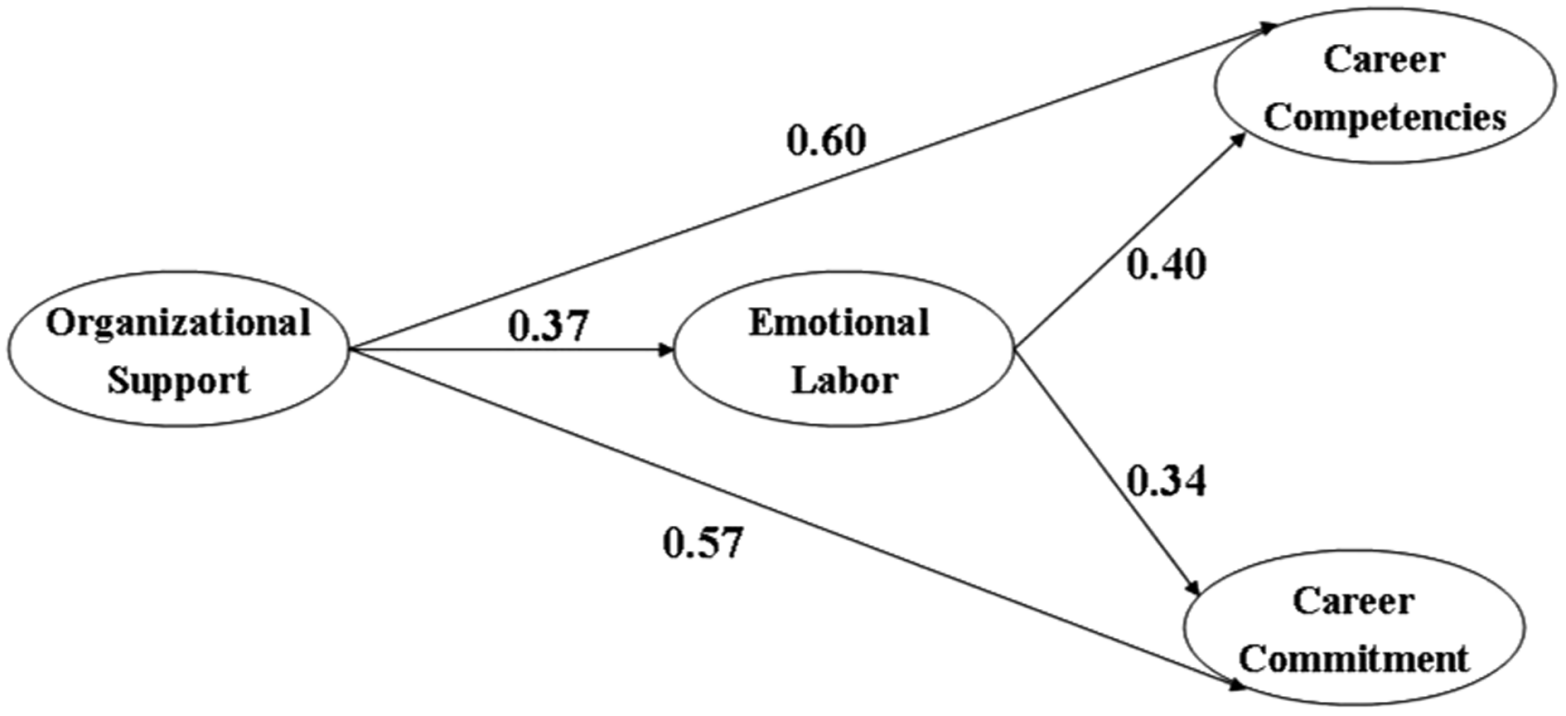
Estimated measurement paths.

Lastly, the structural model was tested to evaluate the hypothesized relationships among the variables. The model fit indices were reported as follows: *x*^2^ = 1103.26, *df* = 282, CFI = 0.93, GFI = 0.91, RMSEA = 0.07, thus representing a fit to the data. It was demonstrated by the results that all structural path estimates reported statistical significance (as indicated by C.R. >1.91), meeting the standards specified by Hu and Bentler (1998), and therefore could be validated. The path coefficient value and significant level, which is delineated in Figure 2, illustrate positive and significant structural paths. Thus, with all the direct positive relationships confirmed, all of the proposed hypotheses were proved to be statistically significant and thereby supported. Specifically, the mediating function of emotional labor was consolidated through the Sober Test, with positive indirect effect coefficients (0.36, 0.39) and *t*-values (6.73, 7.09). Accordingly, it could be concluded that emotional labor had mediated the effects of organizational support on career competences and career commitment.

Besides, multiple regression analyzes were conducted to scrutinize the specific effects of the respective dimensions of emotional labor and cross-examined with the criterion as suggested by Hair et al. (2014). It was revealed that organizational support exerted a positive effect on the construct of genuine acting (*β* = 0.63, *p* < 0.001), a negative effect on the construct of surface acting (*β* = −0.20, *p* < 0.001), and little effects on the dimension of deep acting. On the other hand, career competences were discovered to be positively influenced by deep acting and genuine acting (*β* = 0.38 and 0.40 respectively), while little influenced by surface acting. Finally, the impacts of emotional labor dimensions on career commitment were similar to those on career competences, with deep acting and genuine acting indicating positive influences (*β* = 0.27 and 0.31 respectively) and surface acting reporting negative effects (*β* = −0.24).

## Discussion

This study, through comprehensive modeling and systematic analyzes, investigates the relationships among emotional labor and significant human resource management constructs of organizational support, career competences and career commitment. As has been illustrated by the SEM results, there exist positive effects of emotional labor on career competences and career commitment, with organizational support exerting the mediating role on such influences. Findings of this study, therefore, present a complementary perspective on the mechanism of emotional labor in accounting for the organizational behaviors of the employee, from upon which valuable academic as well as practical implications can be derived.

Theoretically, this study makes it due contributions to literature by evaluating the impacts of emotional labor in a managerial context, thereby enriching current literature which is dominant with employee-centered elaborations on emotional labor. Results of this study, as evidenced by the confirmations of hypotheses 1–3, have consolidated the considerable influences of emotional labor on major career management constructs of the employee, which empirically echoes the articulations of Pizam (2004) and Kim (2008) that emotion management has become one of the lifelines of the tourism and hospitality industry. The tourism and hospitality industry is one of the most emotion-intensive in the service sector, and has to adapt itself constantly to a more and more diversified and demanding market, requirements for more personalized and all-around services, as well as integrations of ‘the human factor’ with latest technological advances (Xu, 2007). All of such challenges, when complicated by the emergency of the generations Y and Z into the workforce who themselves are characterized by self-centered personalities and unscrupulous expressions of genuine emotions, highlight the significance of management of emotional labor in addressing the needs of the customers as well as promoting psychological well-being of the employees. As emotional labor has been found from examinations of hypotheses 2–3 to positively contribute to career competences and career commitment of the employee, it can be assumed here that emotional labor may serve as an empowering force that can inspire the motivations for and identification of the employee with his/her career, just as has been suggested by Pugliesi (1999). In this sense, emotional labor can be articulated as a facilitator of the positive attitudinal psychological dynamics of the employee, particularly when the genuine emotions of the employee are consistent with those expected by the job.

With regard to the specific impacts of the three dimensions of emotional labor, the contrast in the effects of surface, deep and genuine acting in enhancing the level of career competences and career commitment of the employee is consistent with the results of previous studies reporting diversified influences of different categories of emotional labor on major consequence factors (Yan et al., 2012). In the condition of surface acting, the employee fakes an organizational identity which is detached from his or her authentic feelings, while deep acting mobilizes the employee’s willpower to attach to his or her organizational identity, with genuine acting representing the congruity between the employee’s own feelings and those required by the job. In this sense, an ascending order of mental activeness and consistency is observed in surface, deep and genuine acting, which would consequently shape the level of attitudinal inclinations of the employee toward his or her career. As has been put by Chu and Murrmann (2006), emotional labor is a contagion that closely regulates the direction and intensity of other significant psychological traits. This is further supported by the natures of career competencies and career commitment, which are based on the subjective perceptions and evaluations made by the employee, are inherently underlined by emotion-laden foundations (Liu et al., 2004). When employees identifies with the work emotionally, they would be more motivated to accrue competencies which would better facilitate their career path, and be more loyal and committed to the career. This is especially the case under the scenarios of demanding job responsibilities or tough customers, when positive emotional labor would sustain and enhance the psychological attachment of the employee to the organization and prevent burnout and quitting (Brotheridge and Lee, 2003).

## Implications

While it has been argued by some studies that emotional labor is deeply embed into the realm of personalities and quite immune from external influences (Chu et al., 2011; Gursoy et al., 2011), there still exists vast potential for the realization of an emotional-fit between the employee and the organization, so that the positive effects of emotional labor can be optimized. This is best demonstrated by the findings of this study on the mediating roles assumed by organizational support on emotional labor as revealed by testing of hypotheses 4 and 5. In a sense, organizational support can be regarded as the emotional rewards offered by the organization that aim to arouse corresponding emotional identifications and concrete behaviors from the employee. In the hospitality industry, which is already crippled by comparatively low salaries and material benefits, emotional supports from the organization in the forms of care, concern, appreciation, etc. would not only induce due social exchange responses from the employee, but also provide a reliable emotional harbor when the employee encounters problems and challenges. Although this study does not confirm a determining role of organizational support, it does reveal a unique and valuable channel through which the organization can strike an emotional resonance with employees.

Practically, pertinent strategies and solid measures can be proposed and implemented to best utilize emotional labor in improving service quality as well as enhancing customer experiences in the hospitality industry. With modern consumptions strongly underscored by experiences of the product or service by customers, close attention to the emotional health of the employees whose feelings and expressions are integral to customer experiences will definitely be a pertinent demonstration of agile organizational leadership that heralds employee attachment and career endorsement, especially in face of uncertain challenges like the pandemic. Firstly, sessions on emotional management and adjustment should be highlighted in the training and career development programs, with the objective of integrating the genuine emotions of the employee into their work. Meanwhile, based upon the dimensionality of emotional labor explored by the research findings, workshops featuring role plays should be held incorporating perspective of the psychology of customers, based upon which the deep acting tactics and approaches which would be most effective can be customized and tailor-made. Experiences from the performing arts sector, which have already been applied in the theme park industry, seem to offer tenable references here with the emphasis of ‘theatrification’ of services (Ellis and Rossman, 2008). Moreover, at the senior level, corresponding to the research findings from investigations of hypotheses 1–3, the organizational culture should be modified correspondingly so that a better employee-organization fit in emotional terms can be achieved. This would be of particular merits in soliciting the younger generations of workforce featured with strong predilection for employers which they identify with themselves (Kim, 2008). At last, effective measures should be devised and implemented to substantiate the organizational support for the employees, thus creating amicable contexts for the utilization of the employees’ emotional labor.

## Limitation and future research

Despite the efforts by this study to comprehensively and systematically examine emotional labor on the managerial spectrum, it is not without limitations to be addressed by future studies. Firstly, the scales measuring the investigated constructs can be further refined and adjusted to account for the mechanisms of emotional labor in a more tenable manner. There might be redundant items that need to be disposed of, and the scales may not be exhaustive. Hence, future research is guaranteed that enriches and enhances the current scales more rigorously, succinctly and in-depth. Especially, a mixed methodology approach can be selected, soliciting other key indices of emotional labor such as frequency, intensity as well as qualitative data, which can then be triangulated to generate new insights into emotional labor in the tourism and hospitality industry. In addition, taking cognizance of the variety of paradigms in deliberations on emotional labor, this study only avails a partial examination of the effects of emotional labor from a limited perspective. Future research may integrate other significant viewpoints and concepts like working experiences, seniority, personality, as well as cultural values in investigation, so as to facilitate a thorough understanding of the mechanism through which emotional labor influences the attitudinal and behavioral traits of the employee. Thirdly, since this study only focuses on the frontline staff working at luxury hotels, there exists the inherent issue of generalizability. Future studies which encompass a broader range of staff origins—preferably with cross-sectional evaluations of employees from different managerial levels—would definitely contribute to a more in-depth understanding of emotional labor in the tourism and hospitality industry. A unique area of considerable interest would be the exploration of emotional labor in employee-to-employee relationships.

## Data availability statement

The raw data supporting the conclusions of this article will be made available by the authors, without undue reservation.

## Ethics statement

Ethical review and approval was not required for the study on human participants in accordance with the local legislation and institutional requirements. The patients/participants provided their written informed consent to participate in this study.

## Author contributions

YH: conceptualization. WT: data analysis. XW: literature review. LZ: data interpretation. QY: discussion and conclusion. All authors contributed to the article and approved the submitted version.

## Conflict of interest

The authors declare that the research was conducted in the absence of any commercial or financial relationships that could be construed as a potential conflict of interest.

## Publisher’s note

All claims expressed in this article are solely those of the authors and do not necessarily represent those of their affiliated organizations, or those of the publisher, the editors and the reviewers. Any product that may be evaluated in this article, or claim that may be made by its manufacturer, is not guaranteed or endorsed by the publisher.
